# The effectiveness and safety of ofatumumab for the treatment of pemphigus vulgaris: a cohort study based on a registry database

**DOI:** 10.3389/fimmu.2025.1537334

**Published:** 2025-07-25

**Authors:** Xiwen Zhang, Yiyi Wang, Ping Tan, Xingli Zhou, Yue Xiao, Xun Feng, Jishu Li, Mintong Wei, Min Zou, Gyeongah Kim, Lu Jiang, Xiaohong Li, Jinqiu Wang, Mi Wang, Wei Li

**Affiliations:** ^1^ Department of Dermatology & Rare Disease Center, West China Hospital, Sichuan University, Chengdu, Sichuan, China; ^2^ West China School of Nursing, Sichuan University, Chengdu, Sichuan, China; ^3^ Department of Outpatient, West China Hospital, Sichuan University, Chengdu, Sichuan, China

**Keywords:** pemphigus, therapy, ofatumumab, CD20 monoclonal antibody, B cell depletion, biologics

## Abstract

**Background:**

Ofatumumab, a fully human anti-CD20 monoclonal antibody administered subcutaneously and indicated for multiple sclerosis, might theoretically be effective for patients with pemphigus vulgaris (PV).

**Objective:**

To evaluate the effectiveness and safety of ofatumumab in patients with PV.

**Methods:**

This cohort study was based on a registry database of autoimmune bullous diseases at West China Hospital (AIBD-WCH), including two groups. One was ofatumumab (OFA) group, involving patients receiving ofatumumab subcutaneous injections (2×20mg, 2 weeks apart) and systemic glucocorticoids with/without immunosuppressant. The glucocorticoids control (GC) group was matched using propensity score matching in a 1:2 ratio based on sex, age and body mass index. Both groups completed regular follow-up for 52 weeks. The primary endpoint was the proportion of patients achieving complete remission during therapy (CRDT) at week 52. Secondary endpoints included maintaining treatment (MT) with daily prednisone doses <0.2 mg/kg/d, relapse rate, the change of pemphigus disease area index and cumulative glucocorticoid doses. Safety results were also collected.

**Results:**

Sixteen and thirty-two patients were included in OFA and GC groups, respectively. At week 52, more patients in OFA group achieved CRDT (31.2% versus 3.12%, *p*=0.012) and MT (68.8% versus 25.0%, *p*=0.009). Furthermore, patients in OFA group took lower cumulative glucocorticoid doses by week 52 (6186 [SD: 1177]mg versus 9317 [SD: 1579]mg, *p*<0.001). A patient in OFA group experienced gastric hemorrhage, which was judged to be unrelated to ofatumumab, while two in GC group developed lung infections.

**Conclusions:**

Ofatumumab combined with glucocorticoids demonstrated favorable effectiveness compared with GC group, without increasing severe adverse events.

## 1 Introduction

Pemphigus vulgaris (PV) is a chronic, life-threatening autoimmune bullous disease (AIBD) characterized by erythema, blisters, and erosions on the mucosa and/or skin (1). Autoreactive B cells play a crucial role in the pathogenesis of PV, which can differentiate into plasma cells that secrete autoantibodies targeting desmoglein (Dsg) 1 and 3, leading to acantholysis and blister formation. Rituximab, a chimeric murine/human monoclonal antibody against CD20 on the surface of B cells, has demonstrated efficacy in patients with PV and is now listed as a first-line treatment for moderate to severe cases (2). Although rituximab has guideline-recommended protocols (2) (2×1000 mg, 2 weeks apart or 4×375 mg/m^2^, 1 week apart), there are ongoing discussions about alternative dosing and frequency options (3–6). Moreover, studies focusing on the long-term prognosis of patients receiving rituximab reported relapse rates of 63%-100% after a single cycle of infusion (7, 8). Other limitations of rituximab include the administration of intravenous infusion, severe adverse events (AEs) and the costs of hospitalization.

Ofatumumab is a fully human monoclonal antibody targeting CD20 administered subcutaneously and indicated for multiple sclerosis (MS) (9). Over the years, it has been reported to be used in various autoimmune diseases, including rheumatoid arthritis, systemic lupus erythematosus and ANCA-associated vasculitis (10–12). Applications of ofatumumab in pemphigus have been reported in several cases including from our center (13, 14). Recently, a cohort study reported that ofatumumab facilitated rapid disease control in patients with pemphigus compared with the conventional group in 36-week observation (15). However, longer-term data on its effectiveness and safety, and the exploration on alternative injection regimen are still needed. Herein, we present a cohort study that lasted for 52 weeks to evaluate the effectiveness and safety of ofatumumab combined with oral glucocorticoids in PV patients. We hope this study can provide foundational knowledge and regimen references for treating PV with ofatumumab.

## 2 Materials and methods

### 2.1 Data source

The data used in this study derived from a registry database: autoimmune bullous diseases at West China Hospital (AIBD-WCH). The database has been including patients diagnosed with AIBD at the Department of Dermatology, West China Hospital since 1 June 2017, with continuous updates in their medical data. In detail, we systematically collected the patients’ demographical and clinical disease characteristics at their first visit and renewed their data at each visit longitudinally. The database was approved by the biomedical research ethics committee of West China Hospital of Sichuan University (Approval number: 2017–241) and conducted in accordance with the declaration of Helsinki. All participants have signed informed consent.

### 2.2 Study design and participants

**Figure 1:**
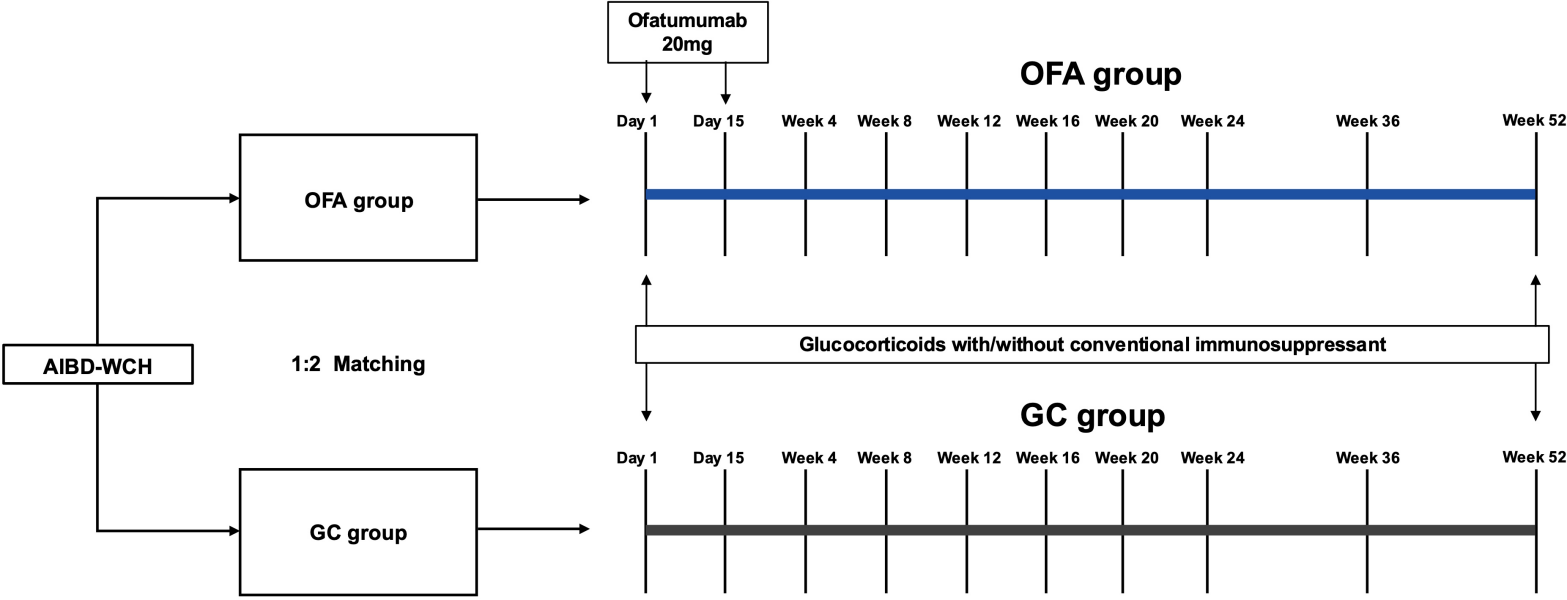
Study design, treatment and follow-up course. OFA group involved patients receiving ofatumumab injections (2×20mg, 2 weeks apart) and systemic glucocorticoids, with or without conventional immunosuppressants. The patients in GC group were 1:2 matched from the AIBD-WCH based on sex, age and BMI. Both groups were regularly followed up for 52 weeks. AIBD-WCH, autoimmune bullous diseases at West China Hospital; OFA group, ofatumumab group; GC group, glucocorticoids group.

This is a cohort study including two groups, the study design is shown in **Figure 1**. Ofatumumab (OFA) group included patients receiving ofatumumab injections and systemic glucocorticoids, with or without conventional immunosuppressants. Inclusion criteria for this group were patients: (i)aged between 18 and 80 years, (ii)with a confirmed diagnosis of PV (based on the clinical presentation, histopathological biopsy, enhancing levels of anti-Dsg1 and/or Dsg3 antibody by enzyme-linked immunosorbent assay, and direct immunofluorescence assay showing immunoglobulin G intercellular deposition of epithelium) (2). Exclusion criteria of OFA group included: (i)known active infections or recent infections which required oral antibiotic treatment within 2 weeks prior to the study, (ii)severe organ dysfunctions, (iii)current malignancies or malignancies within the past 10 years, (iv)pregnancy and breastfeeding or planning to get pregnant, (v)vaccination within 6 weeks prior to the study, (vi)rituximab or other B-cell-targeted therapy within 12 months prior to the study, (vii)plasmapheresis or plasma exchange, immunoadsorption or intravenous immunoglobulins within 8 weeks prior to the study, (viii)refusing written informed consent.

The glucocorticoid control (GC) group included patients with at least one year of regular follow-up in the AIBD-WCH database. In these patients, propensity score matching (PSM) was performed at a 1:2 ratio using nearest-neighbor matching and a caliper of 0.2 without replacement, based on sex, age and body mass index (BMI).

Disease severity was assessed based on an established grading criterion (PDAI: 0–15 for mild, 15–45 for moderate, and ≥45 for severe disease) (16). Patients were considered to have newly diagnosed disease if they had received no more than 1 month of systemic treatments for pemphigus before they were included into the cohort. Patients with systemic treatment over 1 month were considered to have established disease.

### 2.3 Treatment and follow-up

Patients in OFA group received 20mg ofatumumab subcutaneous injection on day 1 and day 15, respectively. Systemic prednisone was initiated at 0.2-1.0 mg/kg/d, with or without conventional immunosuppressants depending on the disease status and severity. Patients in GC group received initial oral prednisone equivalent at a dosage of 0.5-1.0 mg/kg/d, with or without conventional immunosuppressants. The patients were followed up regularly for 52 weeks. The regimens were adjusted according to the disease conditions and laboratory indicators at each visit, and the maintenance dose of ofatumumab was available if necessary.

### 2.4 Outcome measures

The primary endpoint was the proportion of patients who achieved complete remission during therapy (CRDT) at week 52: the absence of new or established lesions while the patient is receiving prednisone equivalent at a dose of 10mg/d or less and/or minimal adjuvant therapy for at least 2 months (2). Secondary endpoints included the proportion of patients who achieved maintaining treatment (MT) at week 52, defined as the absence of new or established lesions with prednisone equivalent tapered to 0.2 mg/kg/d or lower (17), the number of patients experiencing disease relapse, the change of pemphigus disease area index (PDAI) activity score from baseline and cumulative oral glucocorticoids dose over the 52-week observation. Relevant laboratory indicators including serum anti-Dsg1 and anti-Dsg3 antibody levels and absolute CD19+ B cell count of OFA group were assessed regularly. AEs were recorded accurately and evaluated in Common Terminology Criteria for Adverse Events (CTCAE) version 5.0.

### 2.5 Statistical analysis

Continuous variables were presented with measures of central tendency (mean, median) and dispersion (SD, IQR). For normally distributed data, means were compared using the student’s t-test. The Mann-Whitney test was used for non-normally distribution. Categorical variables were described by frequencies and proportions and compared using the Chi-squared test or Fisher’s exact test. Logistic regression was performed to adjust for potential confounders and assess the independent effect of ofatumumab on treatment effectiveness. Linear mixed-effects models were used to analyze repeated measures data, including PDAI scores and anti-Dsg1/3 antibody levels. The significance level was set at 0.05 and all statistical tests were two-sided. R version 4.3.1 and GraphPad Prism 10.0 were used for statistical analyses and illustration.

## 3 Results

### 3.1 Baseline characteristics

In total, 16 patients with PV were included in the OFA group, and 32 patients were included in the GC group, matched by sex, age, and BMI. At baseline, no significant difference was observed in sex, age, BMI, skin/mucosa involvement, severity, PDAI, disease status, duration, and anti-Dsg1/3 antibody levels between the two groups (**Table 1**). The patients in OFA group received lower initial prednisone doses than those in GC group (40.0 [23.8-40.0] mg/d versus 50.0 [40.0-56.2] mg/d, *p*<0.001).

**Table 1:** The comparison of baseline characteristics between two groups.

Characteristics	OFA group (N=16)	GC group (N=32)	*p* value
Sex (%)			0.610
Female	9 (56.2)	14 (43.8)	
Male	7 (43.8)	18 (56.2)	
Age (SD), years	51.1 (12.5)	50.0 (11.5)	0.772
BMI (SD), kg/m^2^	23.8 (3.07)	23.8 (2.87)	0.946
Skin involvement (%)			0.592
No	2 (12.5)	2 (6.2)	
Yes	14 (87.5)	30 (93.8)	
Mucosa involvement (%)			1.000
No	0 (0.00)	1 (3.1)	
Yes	16 (100)	31 (96.9)	
Disease severity^†^ (%)			0.526
mild	4 (25.0)	8 (25.0)	
moderate	11 (68.8)	24 (75.0)	
severe	1 (6.2)	0 (0.0)	
PDAI (IQR)	19.0 (14.2-23.2)	21.0 (15.5-27.0)	0.525
Disease status^‡^ (%)			0.915
Established	11 (68.8)	20 (62.5)	
Newly diagnosed	5 (31.2)	12 (37.5)	
Disease duration (IQR), months	14.5 (10.0-28.0)	12.0 (5.50-12.0)	0.079
Anti-Dsg1 antibody (SD), u/ml	125 (59.2)	126 (54.8)	0.955
Anti-Dsg3 antibody (SD), u/ml	146 (30.6)	151 (49.0)	0.687
Initial glucocorticoid doses^§^ (IQR), mg/d	40.0 (23.8-40.0)	50.0 (40.0-56.2)	<0.001

^†^Disease severity was assessed based on an established grading criterion (PDAI: 0–15 for mild, 15–45 for moderate, and ≥45 for severe disease). ^‡^Patients were considered to have newly diagnosed disease if they had received no more than 1 month of systemic treatments for pemphigus before they were included into the cohort. Patients with systemic treatment over 1 month were considered to have established disease. ^§^Presented with prednisone equivalent; OFA group, ofatumumab 20mg twice in a two-week interval combined with glucocorticoids with/without immunosuppressant; GC group, glucocorticoids with/without immunosuppressant; BMI, Body mass index; PDAI, Pemphigus disease area index; Dsg, desmoglein.

### 3.2 Comparison of outcome indicators between two groups

One patient in OFA group received a third injection at week 32. At week 52, 5 out of 16 patients (31.2%) in OFA group achieved CRDT, compared with 1 out of 32 patients (3.12%) in GC group (*p*=0.012) (**Table 2**). The proportion of patients who achieved MT at week 52 was significantly higher in OFA group than in GC group [11 out of 16 (68.8%) versus 8 of 32 (25.0%), *p*=0.009]. To adjust for potential confounding by baseline prednisone doses, bivariate logistic regressions were performed, focusing on the achievement of CRDT and MT (**Supplementary Tables 1**, **2**). The adjusted OR of ofatumumab treatment was 14.94 (95% CI: 1.24-179.96) for CRDT and 9.65 for MT (95% CI: 1.68-55.46).

**Table 2:** The comparison of outcome indicators between two groups at week 52.

Outcomes	OFA group (N=16)	GC group (N=32)	*p* value
CRDT (%)	5 (31.2)	1 (3.12)	0.012
Time to CRDT (IQR), days	330 (300-360)	–	–
MT (%)	11 (68.8)	8 (25.0)	0.009
Relapse^†^ (%)	0 (0.00)	5 (15.6)	0.154
Cumulative glucocorticoid doses^‡^ (SD), mg	6186 (1177)	9317 (1579)	<0.001

^†^Defined as appearance of 3 or more new lesions in a month that do not heal within 1 week, or the extension of established lesions in a patient who has achieved disease control; ^‡^Presented with prednisone equivalent; OFA group, ofatumumab 20mg twice in a two-week interval combined with glucocorticoids with/without immunosuppressant; GC group, glucocorticoids with/without immunosuppressant; CRDT, Complete remission during therapy; MT, Maintaining treatment.

There was no significant difference in the proportion of patients who experienced relapse between the two groups. By week 52, the mean cumulative prednisone exposure of OFA group was lower compared with that of GC group (6186 [SD: 1177]mg versus 9317 [SD: 1579]mg, *p*<0.001). There was no significant difference in the application or type of conventional immunosuppressants between the two groups.

**Figure 2:**
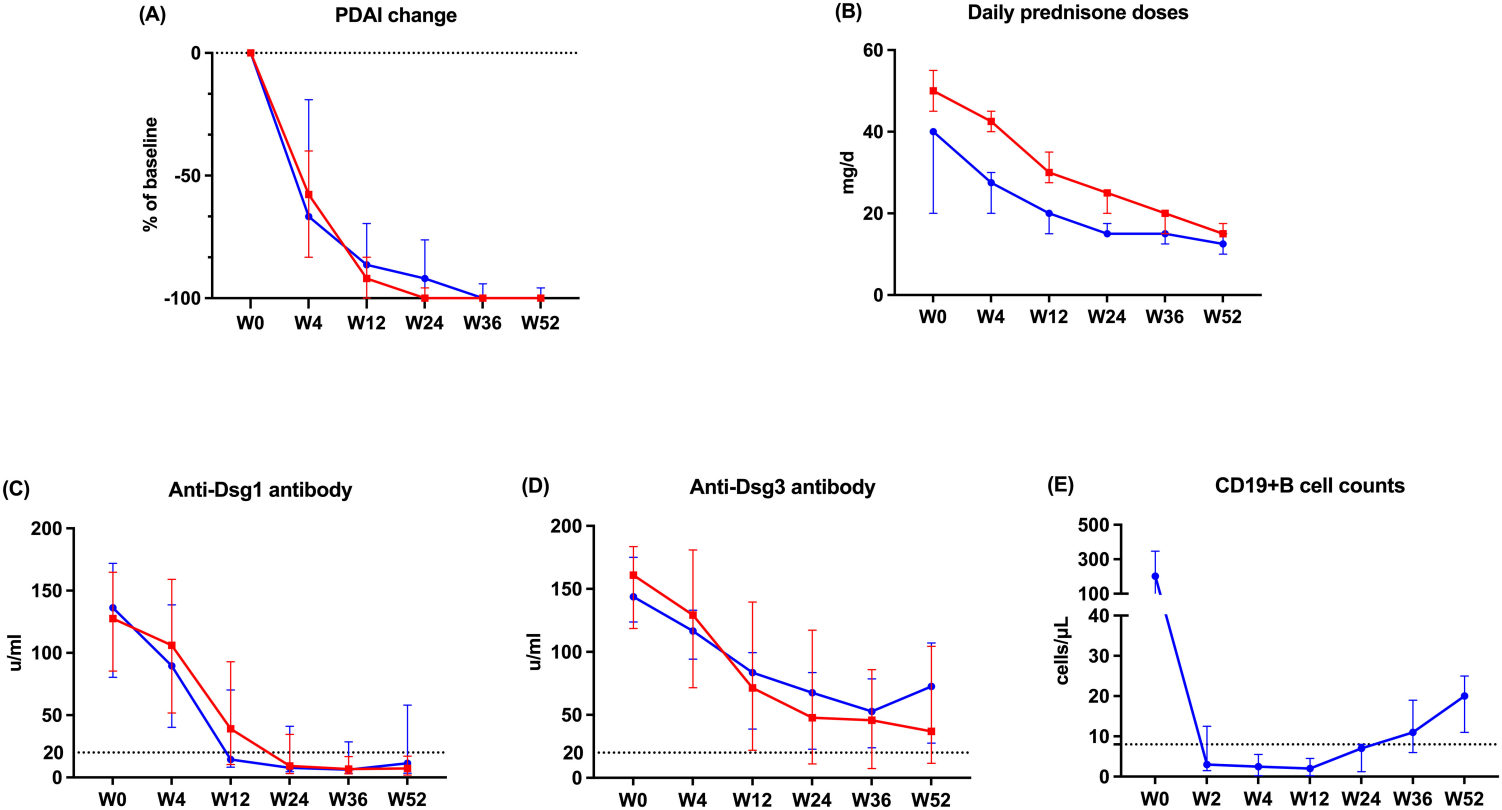
The change of outcome indicators during the study. **(A)** The change of PDAI **(B)** Daily prednisone doses **(C)** Serum anti-Dsg1 antibody level **(D)** Serum anti-Dsg3 antibody level **(E)** Absolute CD19+B cell count. The blue lines represent OFA group. The red lines represent GC group. PDAI, pemphigus disease area index; Dsg, desmoglein.

Over the 52-week observation, both groups showed a steadily decreasing trend in PDAI, with no significant differences in PDAI changes between the two groups at each visit (**Figure 2A**, **Supplementary Table 3**). The OFA group received significantly less daily prednisone doses than the GC group from week 0 to week 52 (**Figure 2B**, **Supplementary Table 4**). **Supplementary Image 1** displays the treating course of a refractory patient in OFA group.

Moreover, we performed exploratory subgroup analysis according to treatment history (**Supplementary Table 5**). In both subgroups, the proportions of patients achieving CRDT and MT in the OFA group show higher trend compared with those of the GC group. Additionally, among the five patients who were newly diagnosed and received ofatumumab as first-line therapy, 2 (40.0%) achieved CRDT, compared with 3 out of 11 (27.3%) among patients with established disease.

### 3.3 Laboratory findings

At each visit, no significant difference in anti-Dsg1/3 antibody levels was found between the two groups (**Figures 2C, D**, **Supplementary Table 3**). Anti-Dsg1 antibody levels decreased from baseline to week 52 in both groups. Anti-Dsg3 antibody of patients in OFA group decreased until week 36 and then started to increase. After the injection of ofatumumab, the absolute CD19+ B cell counts of patients in OFA group decreased rapidly and remained depleted (defined as 8 cells/μL) by week 24. Afterwards, the median CD19+B cell count increased steadily by week 52 (**Figure 2E**).

### 3.4 Adverse events between two groups

Injection-related reactions (IRRs) were observed in 4 patients (25%) within 24 hours after ofatumumab injection. In the OFA group, one patient experienced a grade 3 AE (gastric hemorrhage) 25 days after ofatumumab administration and 30 days after initiation of glucocorticoids, causing hospitalization for 6 days. Given the patient’s history of chronic alcoholic gastritis and the concurrent use of high-dose prednisone (50 mg/d), this AE was assessed unrelated to ofatumumab. Two patients in GC group developed grade 3 AEs (lung infections). The details of all AEs over 52 weeks are provided in **Supplementary Table 6**.

## 4 Discussion

This cohort study, lasting for 52 weeks, shows that the application of ofatumumab (2×20mg, 2 weeks apart) in patients with PV demonstrated better effectiveness compared with the GC group. At week 52, higher proportion of patients in the OFA group achieved CRDT and MT compared with the GC group. Throughout the entire study, the OFA group received lower daily and cumulative glucocorticoid doses than the GC group. No severe AE related to ofatumumab was reported in the OFA group. The subcutaneous administration of ofatumumab is more convenient, accessible, and acceptable for patients with pemphigus compared to the intravenous infusion of rituximab, which typically requires inpatient clinic visits. Furthermore, due to its fully human structure, ofatumumab is theoretically less immunogenic, potentially reducing the risk of adverse effects associated with anti-drug antibody formation.

The proportion of patients achieving CRDT in our study was 31.2%, appearing lower compared with the data reported in previous studies on rituximab, which ranged from 40% to 100% (3, 5, 6, 8, 18, 19). These differences can be attributed to variations in study designs, patient conditions, treatment protocols and glucocorticoid-tapering regimens. In our cohort study conducted in a real-world setting, we adopted a conservative and individualized tapering protocol to minimize the risk of relapse, resulting in a delayed reduction to a prednisone dose of 10mg/kg/d. Therefore, it’s difficult to directly compare the efficacy of ofatumumab and rituximab based on the endpoint of CR. A well-designed head-to-head study would be suitable for this purpose. At week 52, a significantly higher proportion of patients in the OFA group achieved MT compared with the GC group, suggesting a favorable steroid-sparing effect from the perspective of personalized therapy.

In the newly diagnosed subgroup, we observed a trend toward a higher proportion of patients achieving CRDT compared with those with established disease. This finding aligns with the results from rituximab studies, suggesting that early application of ofatumumab might benefit patients with PV (20, 21).

No patient in our study experienced relapse by week 52, which somehow differed from the relapse data from previous studies (for instance, around 25% in the first year with rituximab (3)). Several factors may explain this difference. Firstly, as descripted above, our tapering protocols differed from those in prior studies. We adjusted prednisone doses based on each patient’s disease condition and immune biomarkers at each visit, generally in a lower tapering speed rather than rescue treatment, aiming to reduce the relapse risk. Another reason was the varying distributions of disease severity, a known risk factor for disease relapse (22). In our study, the OFA group consisted primarily of mild to moderate patients (PDAI: 19.0 (14.2-23.2)), while previous studies typically focused on moderate to severe patients. Additionally, we did observe that a refractory patient in the OFA group whose B cell count increased to 61 cells/μL with a high anti-Dsg1 antibody level by week 32. As a result, a third injection of ofatumumab was administered. Following this adjuvant dose, the anti-Dsg1 antibody level decreased rapidly along with the B cell count, and no relapse occurred by week 52.

Reducing oral glucocorticoid doses is a key objective in the development of new therapies for PV. Since baseline, the daily prednisone doses of the OFA group were significantly lower than those in the GC group over the follow-up. Nevertheless, higher proportion of patients in the OFA group achieved CRDT and MT, and the PDAI showed no significant differences at each visit. As a result, patients in the OFA group took lower cumulative prednisone dose compared with the GC group by week 52. These findings align with the results from the Ritux3 trial (3), supporting the robust steroid-sparing effect of ofatumumab.

Plenty of research proposed the association between B cell repopulation and relapse (7, 23, 24). Our data presented increasing trend in B cell count and anti-Dsg3 antibody level since week 24, similar to the data from rituximab (3). It remained below 40 cells/μL by week 52, which is not entirely consistent with the simulated data from the pharmacokinetics study (25). This discrepancy might because of the application of glucocorticoid, inhibiting lymphocytes proliferation. The anti-Dsg3 level also started to elevate at week 36 but remained below the predictive relapse cutoff of 130 U/mL by week 52, consistent with the absence of relapse (23, 26). This could be explained by the recovery of memory B cells secreting different types of IgG subclass, including non-pathogenic antibodies (27). Overall, further data on B cells and auto-antibodies in patients with pemphigus following anti-CD20 therapy are needed to predict prognosis more accurately.

The OFA group demonstrated good tolerability. IRRs occurred in 25% of patients, consistent with the 24.1% reported in the ASCLEPIOS II trial in MS (9), and similar with the 22% of infusion related reactions observed in the RCT of rituximab in pemphigus (19). One severe AE (gastric hemorrhage) was observed in the OFA group (6.3%), versus 22% observed in the safety data for 52 weeks from rituximab (19). The most common AEs in the OFA group included elevation of liver enzymes and gastrointestinal disorders, likely related to the use of conventional immunosuppressants and glucocorticoids. Whereas, the most frequent AEs reported in the phase III RCT in MS include IRRs, nasopharyngitis, headache, upper respiratory tract infection and urinary tract infection (9). The difference in AEs between the OFA group in our study and phase III RCT in MS may lie on GC application. In addition, the highest AE in GC group was osteoporosis, which was not reported in the OFA group, suggesting an encouraging protective effect of ofatumumab against glucocorticoid-induced side effects.

The main limitation of this study is the small sample size, especially the limited number of severe pemphigus, confining further stratified analysis. The 52-week follow-up also constrains the assessment of long-term outcomes.

In conclusion, this study suggests ofatumumab is well-tolerant, effective and convenient for patients with PV, showing favorable steroid-sparing effects in the entire treatment course. The feature of subcutaneous administration enables ofatumumab a potentially convenient option for patients with PV. Further studies are needed to explore the optimal timing of adjuvant ofatumumab injection. Furthermore, prospective head-to-head trials can be conducted to compare the efficacy of ofatumumab and rituximab in patients with PV.

## Data Availability

The raw data supporting the conclusions of this article will be made available by the authors, without undue reservation.

## Supplementary material

Supplementary Figure 1 Clinical pictures of a refractory patient in OFA group. PDAI at week 0: 23, PDAI at week 4: 26 (experienced COVID-19 infection), PDAI at week 12: 7, PDAI at week 24: 5, PDAI at week 52: 3.

## References

[1] Kasperkiewicz M Ellebrecht CT Takahashi H Yamagami J Zillikens D Payne AS. Pemphigus. Nat Rev Dis Primers. (2017) 3:17026. doi: 10.1038/nrdp.2017.26, PMID: 28492232 PMC5901732

[2] Murrell DF Peña S Joly P Marinovic B Hashimoto T Diaz LA. Diagnosis and management of pemphigus: Recommendations of an international panel of experts. J Am Acad Dermatol. (2020) 82:575–85.e1. doi: 10.1016/j.jaad.2018.02.021, PMID: 29438767 PMC7313440

[3] Joly P Maho-Vaillant M Prost-Squarcioni C Hebert V Houivet E Calbo S. First-line rituximab combined with short-term prednisone versus prednisone alone for the treatment of pemphigus (Ritux 3): a prospective, multicentre, parallel-group, open-label randomised trial. Lancet. (2017) 389:2031–40. doi: 10.1016/S0140-6736(17)30070-3, PMID: 28342637

[4] Zhou X Zhan T Xu X Lan T Hu H Xia D. The efficacy and safety of low-dose rituximab in the treatment of pemphigus vulgaris: a cohort study. J Dermatolog Treat. (2024) 35:1:2302071. doi: 10.1080/09546634.2024.2302071, PMID: 38247364

[5] Kanwar AJ Vinay K Sawatkar GU Dogra S Minz RW Shear NH. Clinical and immunological outcomes of high- and low-dose rituximab treatments in patients with pemphigus: a randomized, comparative, observer-blinded study. Br J Dermatol. (2014) 170:1341–9. doi: 10.1111/bjd.12972, PMID: 24640990

[6] Zhang J Huang X Zhang Z Zhao P Chen W Liang Y. Clinical observation of different doses of rituximab for the treatment of severe pemphigus: A single-center prospective cohort study. J Am Acad Dermatol. (2023) 88:500–2. doi: 10.1016/j.jaad.2022.06.1187, PMID: 35779637

[7] Saleh MA. A prospective study comparing patients with early and late relapsing pemphigus treated with rituximab. J Am Acad Dermatol. (2018) 79:97–103. doi: 10.1016/j.jaad.2018.01.029, PMID: 29408700

[8] Shimanovich I Baumann T Schmidt E Zillikens D Hammers CM. Long-term outcomes of rituximab therapy in pemphigus. J Eur Acad Dermatol Venereol. (2020) 34:2884–9. doi: 10.1111/jdv.16561, PMID: 32367562

[9] Hauser SL Bar-Or A Cohen JA Comi G Correale J Coyle PK. Ofatumumab versus teriflunomide in multiple sclerosis. N Engl J Med. (2020) 383:546–57. doi: 10.1056/NEJMoa1917246, PMID: 32757523

[10] Kurrasch R Brown JC Chu M Craigen J Overend P Patel B. Subcutaneously administered ofatumumab in rheumatoid arthritis: A phase I/II study of safety, tolerability, pharmacokinetics, and pharmacodynamics. J Rheumatol. (2013) 40:1089–96. doi: 10.3899/jrheum.121118, PMID: 23729801

[11] Masoud S McAdoo SP Bedi R Cairns TD Lightstone L. Ofatumumab for B cell depletion in patients with systemic lupus erythematosus who are allergic to rituximab. Rheumatology. (2018) 57:1156–61. doi: 10.1093/rheumatology/key042, PMID: 29562252

[12] McAdoo SP Bedi R Tarzi R Griffith M Pusey CD Cairns TD. Ofatumumab for B cell depletion therapy in ANCA-associated vasculitis: a single-centre case series. Rheumatology. (2016) 55:1437–42. doi: 10.1093/rheumatology/kew199, PMID: 27094598 PMC4957674

[13] Zhang X Xiao Y Li X Wang J Zhou X Wang Y. Ofatumumab subcutaneous injection successfully treated patients with pemphigus vulgaris relapse post rituximab. J Dermatol. (2024) 51:1026–30. doi: 10.1111/1346-8138.17108, PMID: 38293719

[14] Klufas DM Amerson E Twu O Clark L Shinkai K. Refractory pemphigus vulgaris successfully treated with ofatumumab. JAAD Case Rep. (2020) 6:734–6. doi: 10.1016/j.jdcr.2020.05.034, PMID: 32715065 PMC7369462

[15] Mao J Bao S Chen Y Zhuang Z Chen W Li G. Evaluation of combination therapy with ofatumumab and systemic corticosteroids for pemphigus: A multi-centre cohort study. Acad Dermatol Venereol. (2024) 39(3):e244-7. doi: 10.1111/jdv.20245, PMID: 38994899

[16] Boulard C Duvert Lehembre S Picard-Dahan C Kern JS Zambruno G Feliciani C. Calculation of cut-off values based on the Autoimmune Bullous Skin Disorder Intensity Score (ABSIS) and Pemphigus Disease Area Index (PDAI) pemphigus scoring systems for defining moderate, significant and extensive types of pemphigus. Br J Dermatol. (2016) 175:142–9. doi: 10.1111/bjd.14405, PMID: 26800395

[17] Committee for Guidelines for the Management of Pemphigus Disease Amagai M Tanikawa A Shimizu T Hashimoto T Ikeda S. Japanese guidelines for the management of pemphigus. J Dermatol. (2014) 41:471–86. doi: 10.1111/1346-8138.12486, PMID: 24909210

[18] Kushner CJ Wang S Tovanabutra N Tsai DE Werth VP Payne AS. Factors associated with complete remission after rituximab therapy for pemphigus. JAMA Dermatol. (2019) 155:1404–9. doi: 10.1001/jamadermatol.2019.3236, PMID: 31642878 PMC6813574

[19] Werth VP Joly P Mimouni D Maverakis E Caux F Lehane P. Rituximab versus mycophenolate mofetil in patients with pemphigus vulgaris. N Engl J Med. (2021) 384:2295–305. doi: 10.1056/NEJMoa2028564, PMID: 34097368

[20] Tedbirt B Maho-Vaillant M Houivet E Mignard C Golinski ML Calbo S. Sustained remission without corticosteroids among patients with pemphigus who had rituximab as first-line therapy: follow-up of the ritux 3 trial. JAMA Dermatol. (2024) 160:290–6. doi: 10.1001/jamadermatol.2023.5679, PMID: 38265821 PMC10809134

[21] Nosrati A Mimouni T Hodak E Gdalevich M Oren-Shabtai M Levi A. Early rituximab treatment is associated with increased and sustained remission in pemphigus patients: A retrospective cohort of 99 patients. Dermatol Ther. (2022) 35:e15397. doi: 10.1111/dth.15397, PMID: 35194896

[22] Mignard C Maho-Vaillant M Golinski ML Balayé P Prost-Squarcioni C Houivet E. Factors associated with short-term relapse in patients with pemphigus who receive rituximab as first-line therapy: A *post hoc* analysis of a randomized clinical trial. JAMA Dermatol. (2020) 156:545–52. doi: 10.1001/jamadermatol.2020.0290, PMID: 32186656 PMC7081151

[23] Albers LN Liu Y Bo N Swerlick RA Feldman RJ. Developing biomarkers for predicting clinical relapse in pemphigus patients treated with rituximab. J Am Acad Dermatol. (2017) 77:1074–82. doi: 10.1016/j.jaad.2017.07.012, PMID: 28927663

[24] Feldman RJ Christen WG Ahmed AR. Comparison of immunological parameters in patients with pemphigus vulgaris following rituximab and IVIG therapy: Immunological parameters in patients with PV treated with rituximab and IVIG. Br J Dermatol. (2012) 166:511–7. doi: 10.1111/j.1365-2133.2011.10658.x, PMID: 21967407

[25] Yu H Graham G David OJ Kahn JM Savelieva M Pigeolet E. Population pharmacokinetic–B cell modeling for ofatumumab in patients with relapsing multiple sclerosis. CNS Drugs. (2022) 36:283–300. doi: 10.1007/s40263-021-00895-w, PMID: 35233753 PMC8927028

[26] Abasq C Mouquet H Gilbert D Tron F Grassi V Musette P. ELISA testing of anti–desmoglein 1 and 3 antibodies in the management of pemphigus. Arch Dermatol. (2009) 145:529–35. doi: 10.1001/archdermatol.2009.9, PMID: 19451496

[27] Golinski ML Lemieux A Maho-Vaillant M Barray M Drouot L Schapman D. The diversity of serum anti-DSG3 IgG subclasses has a major impact on pemphigus activity and is predictive of relapses after treatment with rituximab. Front Immunol. (2022) 13:849790. doi: 10.3389/fimmu.2022.849790, PMID: 35371083 PMC8965561

