# Supplementary figures and images for: The effectiveness and safety of ofatumumab for the treatment of pemphigus vulgaris: a cohort study based on a registry database
Source: Front Immunol. 2025 Jul 25;16:1537334. doi: 10.3389/fimmu.2025.1537334 (PMC12331720; doi:10.3389/fimmu.2025.1537334)

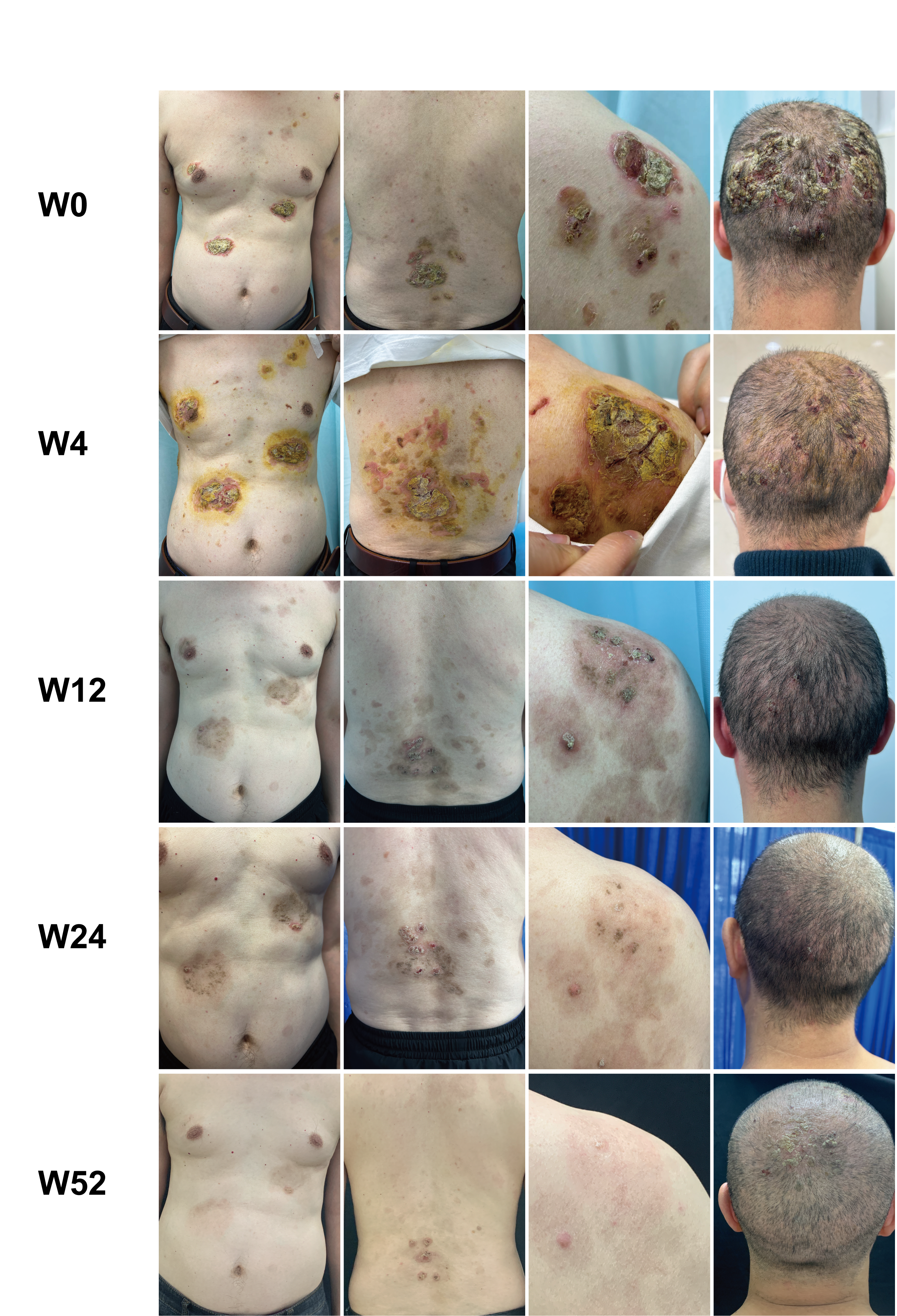

Supplement: Supplementary Figure 1 — Clinical pictures of a refractory patient in OFA group. PDAI at week 0: 23, PDAI at week 4: 26 (experienced COVID-19 infection), PDAI at week 12: 7, PDAI at week 24: 5, PDAI at week 52: 3. [file Image1.tif]
