# Supplementary material for: The effectiveness and safety of ofatumumab for the treatment of pemphigus vulgaris: a cohort study based on a registry database
Source: Front Immunol. 2025 Jul 25;16:1537334. doi: 10.3389/fimmu.2025.1537334 (PMC12331720; doi:10.3389/fimmu.2025.1537334)
Supplement: Supplementary file 2 [file Table1.docx]

Supplementary Table 1. Bivariate logistic regression for CRDT

| Variables | Adjusted OR | 95%CI | p values |
| --- | --- | --- | --- |
| Treatment group |  |  |  |
| GC group | Reference | - | **-** |
| OFA group | 14.94 | 1.24-179.96 | 0.033 |
| Initial prednisone doses | 1.00 | 0.93-1.08 | 0.912 |

GC group: glucocorticoids with/without immunosuppressant; OFA group: ofatumumab 20mg twice in a two-week interval combined with glucocorticoids with/without immunosuppressant; CRDT: Complete remission during therapy
