# Supplementary material for: The effectiveness and safety of ofatumumab for the treatment of pemphigus vulgaris: a cohort study based on a registry database
Source: Front Immunol. 2025 Jul 25;16:1537334. doi: 10.3389/fimmu.2025.1537334 (PMC12331720; doi:10.3389/fimmu.2025.1537334)
Supplement: Supplementary file 3 [file Table2.docx]

Table S2. Bivariate logistic regression for MT

| Variables | Adjusted OR | 95%CI | p values |
| --- | --- | --- | --- |
| Treatment group |  |  |  |
| GC group | Reference | - | - |
| OFA group | 9.65 | 1.68-55.46 | 0.011 |
| Initial prednisone doses | 0.97 | 0.92-1.03 | 0.322 |

GC group: glucocorticoids with/without immunosuppressant; OFA group: ofatumumab 20mg twice in a two-week interval combined with glucocorticoids with/without immunosuppressant; MT: Maintaining treating.
