# Supplementary material for: The effectiveness and safety of ofatumumab for the treatment of pemphigus vulgaris: a cohort study based on a registry database
Source: Front Immunol. 2025 Jul 25;16:1537334. doi: 10.3389/fimmu.2025.1537334 (PMC12331720; doi:10.3389/fimmu.2025.1537334)
Supplement: Supplementary file 7 [file Table6.docx]

Supplementary Table 6. Adverse events between two groups

|  | OFA group (N=16) | GC group (N=32) |
| --- | --- | --- |
|  | Number of patients (%) | |
| Injection-related reactions^†^ | 4(25.0) | - |
| Dizziness | 2(12.5) |  |
| Fever | 2(12.5) |  |
| Fatigue | 2(12.5) |  |
| Injection site edema | 1(6.3) |  |
| Joint pain | 1(6.3) |  |
| Lung infection |  | 2(6.3) |
| Epididymitis |  | 1(3.1) |
| Conjunctivitis | 2(12.5) | 1(3.1) |
| Folliculitis |  | 3(9.4) |
| Superficial mycoses | 1(6.3) | 4(12.5) |
| Viral infection | 2(12.5) | 5(15.6) |
| Epstein-Barr virus | 1(6.3) |  |
| Herpes simplex |  | 2(6.3) |
| Verruca vulgaris | 1(6.3) |  |
| Herpes zoster |  | 3(9.4) |
| Elevation of Liver enzyme | 5(31.3) | 1(3.1) |
| Gastrointestinal disorders | 4(25.0) | 1(3.1) |
| Chronic gastritis | 2(12.5) | 1(3.1) |
| Gastric hemorrhage | 1(6.3) |  |
| Duodenal ulcer | 1(6.3) |  |
| Hyperlipidemia |  | 1(3.1) |
| Steroid acne | 1(6.3) | 1(3.1) |
| Osteoporosis |  | 9(28.1) |
| Cataract |  | 3(9.4) |
| Insomnia |  | 3(9.4) |
| Urticaria | 1(6.3) |  |
| Vitiligo | 1(6.3) | 1(3.1) |

^†^Defined as reactions/symptoms occurring within 24 hours after injection; OFA

group: ofatumumab 20mg twice in a two-week interval combined with glucocorticoids with/without immunosuppressant; GC group: glucocorticoids with/without immunosuppressant.
